# Behaviour change techniques used in lifestyle interventions that aim to reduce cancer-related fatigue in cancer survivors: a systematic review

**DOI:** 10.1186/s12966-023-01524-z

**Published:** 2023-10-13

**Authors:** Judith de Vries-ten Have, Renate M. Winkels, Ellen Kampman, Laura H.H. Winkens

**Affiliations:** 1 Division of Human Nutrition and Health, Nutrition and Disease Chair Group, Wageningen University and Research, Stippeneng 4, 6708 WE Wageningen, The Netherlands; 2 Consumption and Healthy Lifestyles Chair Group, Wageningen University and Research, Hollandseweg 1, 6706 KN Wageningen, The Netherlands

**Keywords:** Diet, Exercise, Physical activity, Behavioural theories, Behaviour change taxonomy

## Abstract

**Background:**

Lifestyle interventions that target dietary and/or physical activity behaviours may impact cancer-related fatigue in cancer survivors. Changing lifestyle may be especially difficult for cancer survivors suffering from cancer-related fatigue. To increase effectiveness of lifestyle interventions, behaviour change techniques (BCTs) can be applied. The aim of this review is to systematically describe which BCTs are applied in lifestyle interventions targeting cancer-related fatigue among cancer survivors who finished primary treatment.

**Methods:**

PubMed, Scopus, PsycINFO, Cochrane Library and Web of Science were searched to identify randomised controlled trials (RCTs) of dietary and/or physical activity interventions targeting cancer-related fatigue in cancer survivors. The BCT taxonomy was used to code the BCTs that were applied in those interventions. BCTs that were reported in at least 25% of effective interventions were indicated as ‘promising BCT’, but only retained this classification when these BCTs were present in less than 25% of ineffective interventions.

**Results:**

Twenty-nine RCTs were identified, of which 17 were effective in reducing cancer-related fatigue. The most frequently applied BCTs were Goal setting (behaviour), Instruction on how to perform the behaviour, Demonstration of the behaviour, Behavioural practice/rehearsal, and Credible Source. The BCT ‘Generalisation of the target behaviour’ was identified as promising. These results should be interpreted with caution as only three studies screened their participants on level of cancer-related fatigue and most studies focused only on physical activity. Furthermore, many studies did not include a measure for actual behaviour change and had no follow-up period after the intervention ended.

**Conclusions:**

There is a need for studies that screen their participants on level of cancer-related fatigue and a need for studies that focus more on dietary behaviours as a possible intervention to reduce fatigue. Also, studies should include follow-up timepoints after the interventions ends to examine long-term behaviour change. Future lifestyle interventions should describe interventions in detail to allow for easier coding of BCTs, and report on actual behaviour change following the intervention. Interventions may apply the BCT ‘Generalisation of the target behaviour’ to incorporate lifestyle behaviours in daily life. This may increase the chance that interventions will effectively reduce cancer-related fatigue.

**Supplementary Information:**

The online version contains supplementary material available at 10.1186/s12966-023-01524-z.

## Background

Many cancer survivors experience long-term side effects after treatment [1]. Cancer-related fatigue is listed as one of the most reported, distressing and severe problems [2, 3], affecting roughly 40% of cancer survivors [4]. Cancer-related fatigue (hereafter referred to as ‘fatigue’) is defined as: ‘’a distressing, persistent, subjective sense of physical, emotional, and/or cognitive tiredness or exhaustion related to cancer or cancer treatment that is not proportional to recent activity and interferes with usual functioning’’ [5]. Fatigue impacts health-related quality of life (HRQoL) and may be a risk factor for reduced survival [6].

Some, but not all, intervention studies suggest beneficial effects of dietary and/or physical activity interventions on fatigue, as previously summarized [7, 8]. A possible reason why not all interventions found beneficial effects on fatigue, could be that the interventions were not successful in achieving substantial behaviour change among the participants. Behaviour change interventions are likely to be more effective when they are grounded in theory and when they incorporate relevant behaviour change techniques (BCTs) to target underlying factors (i.e., determinants) of health behaviours [9]. A BCT is defined as a “replicable component of an intervention designed to alter or redirect causal processes that regulate behaviour; that is, a technique is proposed to be an ‘active ingredient” [10]. The identification of previously used BCTs in trials addressing fatigue, and specifically examining the difference in BCTs used in effective versus ineffective studies helps to identify BCTs that can be used in future intervention development to increase effectiveness of a trial [11]. Another reason why an intervention may not find beneficial effects on fatigue may be that the study in which the intervention was tested was of low quality.

Cancer survivors who experience fatigue, may have more difficulty changing behaviour. This could be because of fatigue itself, or because of the many problems associated with fatigue [12]. Problems associated with fatigue are for example treatment-related side effects (e.g., pain, neuropathy), sleep disturbances, psychological distress, and comorbidities [12]. Treatment-related side effects are important barriers for exercise in fatigued breast cancer survivors [12]. And the fatigue itself is an important barrier for physical activity in cancer survivors [13] and breast cancer survivors specifically [14], and for healthy eating [14, 15]. Problems associated with fatigue should be taken into account when designing lifestyle interventions that intervene on dietary and/or physical activity behaviours for diminishing fatigue [12, 16]. Moreover, evidence-based interventions should be selected and personalized following individual needs and wishes [12, 16] to facilitate long-term behaviour change.

The aim of this review is to systematically describe which BCTs are applied in lifestyle interventions (dietary and/or physical activity behaviours) targeting fatigue among cancer survivors who finished primary treatment. Specific sub aims are to (I) Evaluate the effectiveness of the interventions on reducing fatigue and (II) Identify promising BCTs in studies that are effective in reducing fatigue versus studies that are not effective in reducing fatigue.

## Methods

The review was registered in the Prospective Register of Systematic Reviews database (PROSPERO) with registration number: CRD42021261849. In reporting this review, the Preferred Reporting Items for Systematic Reviews and Meta-Analyses (PRISMA) 2020 statement was used [17].

### Eligibility criteria

Papers were included when they: (1) were physical activity interventions, nutrition interventions, psycho-education interventions on physical activity/nutrition or multi-modality interventions with an physical activity or nutrition component [18], (2) reported on fatigue as outcome, (3) included cancer survivors who finished treatment and were not undergoing any treatment, with the exception of hormone therapy when the focus of the intervention was not specifically on improving outcomes in participants undergoing hormone therapy, (4) were randomised controlled trials (RCTs), (5) applied at least one BCT, (6) were written in the English language, (7) were published as full papers in peer-reviewed international journals, and (8) included a no-intervention wait-list, or usual care/activities control group. Control groups that only received information were also included. All cancer types were included as well as any length of intervention. Papers were excluded when they (1) included participants who were < 18 years of age, or (2) were solely mind-body interventions (e.g., yoga and tai chi) [18].

### Information sources

A systematic literature search was conducted from database commencement to May 2023. The search was conducted in the following databases: PubMed, Scopus, PsycINFO, Cochrane Library and Web of Science. For the final included RCTs we also searched and included corresponding protocols and corresponding papers describing the intervention, if available, as those papers often provided more detailed information on BCTs.

### Search strategy

Various synonyms of the following search terms were used in search queries: ‘cancer survivors’, ‘behaviour change techniques’, ‘behaviour change theory’, ‘behaviour determinants’, ‘lifestyle intervention’, ’randomized controlled trial’, ‘nutrition’, ‘diet’, ‘physical activity’, ‘exercise’ and ‘cancer-related fatigue’. The search queries were developed using the Participants, Intervention, Comparison and Outcome (PICO) framework [19]. The search queries per database can be read from Table S1 in the supplementary materials.

### Study papers

#### Screening

Software for systematic reviews (Rayyan) was used to manage papers and data. One author (JdV) conducted the initial search and deleted duplicates. Two authors (JdV & SB) screened the remaining titles and abstracts for eligibility for inclusion. Conflicts were discussed until consensus was reached. For this screening on title and abstract, we also included papers mentioning QoL as outcome, since fatigue is sometimes seen as part of QoL and is therefore often included in questionnaires assessing QoL. Full texts were independently screened by JdV, SB and LW. JdV and SB screened all papers and LW screened roughly 50% of the papers. Any disagreements were solved by discussion. Studies that were ineligible were excluded from the study with reporting the reason of rejection.

#### Data extraction of characteristics of the studies

Data were extracted by one author (JdV). Data that were extracted are: type of study, type of control group, duration of the intervention, country, type of cancer of the participants, screening conditions, sample size, intervention content, fatigue type of outcome (i.e., primary, secondary, not specified/other), questionnaires used for fatigue, and BCTs used. Any uncertainties were discussed with a second reviewer (LW) to reach consensus.

#### Quality assessment

Quality of the studies was independently examined by two assessors (JdV & LC) using the SIGN checklist for RCTs [20]. Each paper was scored on internal validity; details can be found in supplemental Table S1. The scoring options include Yes, No and Can’t say. Due to the nature of the studies, blinding of participants is not possible in lifestyle interventions. Therefore, all studies scored a ‘No’ on blinding, and we did not consider this in the final assessment of study quality. We used the following criteria to give an overall assessment of study quality: High quality were studies that had no or only one ‘Can’t say’; Acceptable quality were studies where there was one ‘No’ and one ‘Can’t say’ or there were two ‘Can’t say’s’; Low quality were studies where there were two till four ‘No’s’; Studies of unacceptable quality were studies that had more than four ‘No’s’. Studies were immediately scored ‘Low quality’ when a ‘No’ or ‘Can’t say’ was scored on either the randomisation or concealment method.

#### Effectiveness of interventions

Studies that assessed fatigue with a sub-scale as part of a larger questionnaire were included when results of that specific fatigue sub-scale were available. Studies were coded as either statistically significant or not statistically significant. Studies were labelled ‘effective’ when the decrease in fatigue from baseline to post-intervention was statistically significantly (P < 0.05) larger in the intervention group than in the control group. When specific domains of fatigue, such as social fatigue or physical fatigue, were only separately assessed and thus no total fatigue score was reported, we coded it as statistically significant when at least one of those domains was statistically significantly different between intervention and control group.

#### Coding of behaviour change techniques

The BCT Taxonomy of 93 hierarchically clustered techniques divided in 16 categories [11], was used to identify BCTs from the intervention groups of included studies. One author (JdV) coded the BCTs of all papers. A second author (LW) extracted BCTs of roughly 50% of the papers. Any conflicts were solved by discussion. For the remaining 50% of the papers, the first author (JdV) presented any uncertainties to the second author (LW) by showing pieces of text containing the possible BCT, after which a conclusion was drawn on whether to include the BCT, change the coding or exclude it.

##### Behaviour change techniques in effective vs. ineffective studies

We investigated whether the use of specific BCTs was an indicator for effectiveness of the intervention in reducing fatigue, which helps to identify ‘promising BCTs’ [21–25]. Currently, there is no gold standard for determining the effectiveness of BCTs, as all methods assessed in a review of Michie et al. [26] had limitations. Therefore, we chose to use a variation of a common methodology mentioned in that review, where one identifies BCTs used in effective studies [26]. We compared these with BCTs used in ineffective studies, to rule out the possibility mentioned by Michie et al. [26] that ineffective BCTs are deemed effective, just because they are part of the intervention package. The frequency and percentages of each BCT was reported for effective versus ineffective interventions. BCTs that were reported in at least 25% of effective interventions were indicated as ‘promising BCT’ in line with two previous reviews [21, 22], but only retained this classification when these BCTs were present in less than 25% of ineffective interventions. Additionally, we assessed promising BCTs when only including the studies of high and acceptable quality.

## Results

**Fig. 1:**
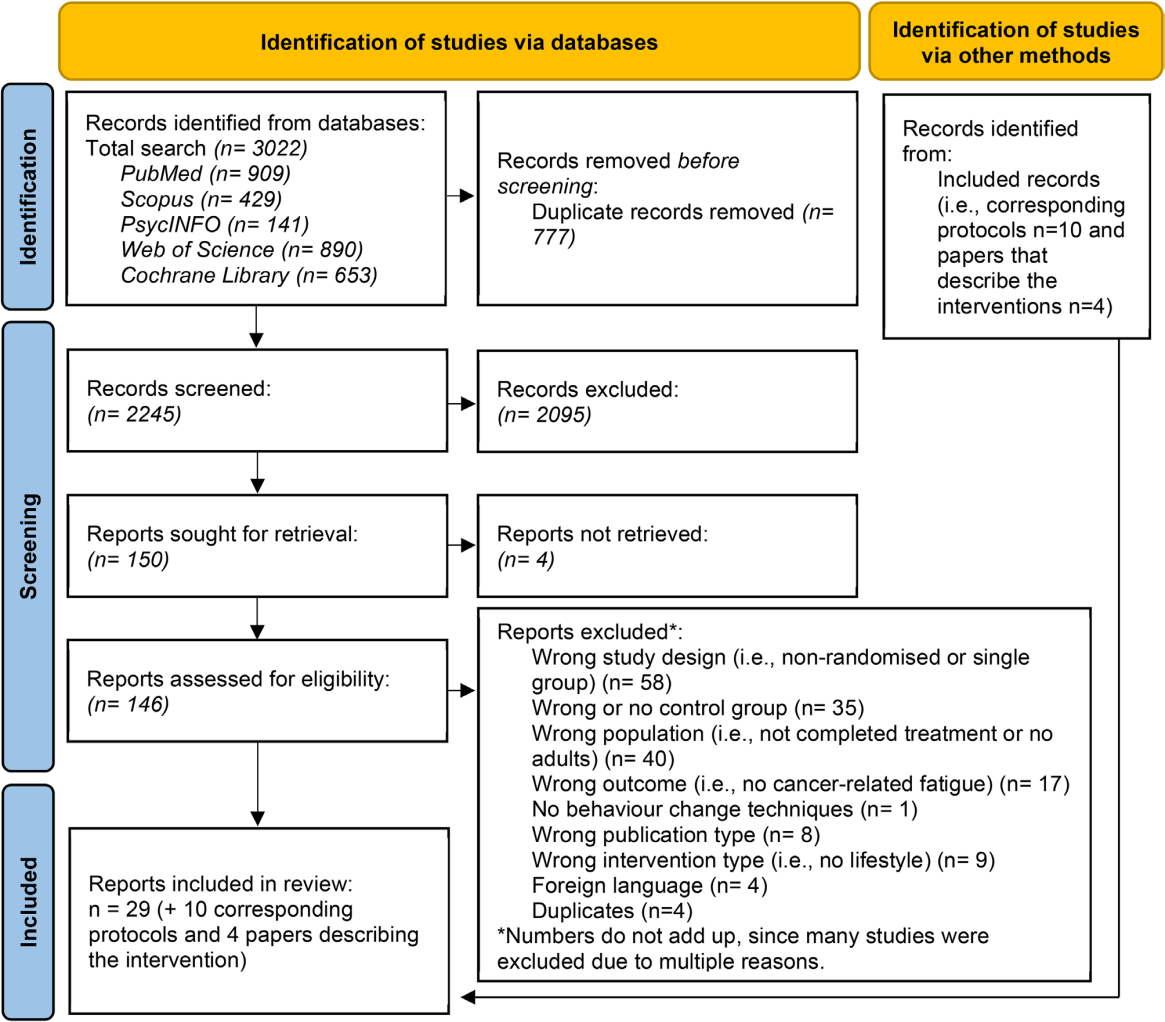
PRISMA flow diagram of article search and selection for the systematic review [17]

Figure 1 displays the PRISMA flow diagram of identified, excluded, and included papers at each step of the review. We extracted a total of 3,022 papers from the five databases, with 2245 papers remaining after removing 777 duplicates. After screening title and abstract, 146 papers were evaluated based on full text assessment, of which 29 papers were included in the study. Furthermore, ten corresponding protocols and 4 corresponding papers describing the intervention were retrieved (Table 1).

**Table 1:** Characteristics of included randomized controlled trials (n = 29﻿)

Study		Intervention		Population			
Reference + *(Additional paper)*	Country	Intervention type	Duration	Cancer type of survivors*	Specific inclusion criteria	Control group	Study size
Adams et al. (2018) [54]	Canada	High-intensity interval training	12 weeks	Testicular	Participants should not perform regular vigorous intensity aerobic exercise	Usual care	63
Bantum et al. (2014) [41]	USA	Web-Based Health Behaviour Change intervention on amongst others mental health, sleep, diet, and exercise	6 weeks (measured only at six months)	Any type *(45.0% breast, 12.8% Endometrium/ Uterine/Ovarian)*		Wait-list / delayed-treatment control	352
Bennett, Lyons, Winters-Stone, Nail, & Scherer (2007) [46]	New Zealand	Motivational interviewing on increasing physical activity	6 months	Any type (76.8% breast)	Participants should be fatigued and inactive	Usual activities	56
Brown et al. (2018) (50) *+* Brown et al. (2016) [76]	USA	Low-dose aerobic exercise and high-dose aerobic exercise	6 months	Colon	≤ 150 min per week of moderate or vigorous intensity physical activity	Usual care	39
Cantarero-Villanueva et al. (2012) [27]	Spain	Multimodal exercise program (8 weeks) + DVD with same exercise program and massage and relaxation techniques, and healthy lifestyle advice	8 weeks	Breast		Usual care	78
Cantarero-Villanueva et al. (2013) [28]	Spain	Deep water aquatic exercise program	8 weeks	Breast	Participants should be fatigued	Usual care	68
Chang et al. (2020) [55]	Taiwan	Nurse-led exercise informatics program, including walking and health education	12 weeks	Esophageal		Usual care	88
Fillion et al. (2008) [31]	Canada	Brief group intervention that combines stress management psychoeducation and physical activity	4 weeks	Breast	Physical activity readiness	Usual care	94
Galiano-Castillo et al. (2016) [29] *+* Galiano-Castillo et al. (2013) [77]	Spain	Telerehabilitation program: internet-based exercise program	8 weeks	Breast		Usual care + information	81
Ghavami & Akyolcu (2017) [37] + Ghavami & Akyolcu (2017) [78]^†^	Iran, Turkey	Supervised aerobic exercises with dietary energy restriction training	24 weeks	Breast	Participants should not be regularly active + BMI > 25 kg/m^2^	Usual care	80
Hagstrom et al. (2016) [33]	Australia	Supervised resistance training	16 weeks	Breast	Participants should be sedentary	Usual care wait-list	39
Hartman et al. (2019) [35] *+* Hartman et al. (2015) [79]	USA	Physical activity intervention	12 weeks	Breast	Participants should be sedentary and have memory / concentration problems	Wait-list wellness contact control	87
Holtdrik et al. (2021) [39] + Holtdirk et al. (2020) [80]	Germany		3 months	Breast		Usual care	363
Kampshoff et al. (2015) [42] *+* Kampshoff et al. (2010) [81]	Netherlands	High-intensity exercise and low-moderate intensity exercise	12 weeks	Breast *(65.3%)*, colon *(18.0%)*, ovarian *(4.3%)*, cervix *(1.3%)*, testis *(1.7%)*, or lymphomas *(9.3%)*		Wait-list	277
Kim et al. (2019) [49] + Lee et al. (2017) [82]	South Korea	Home-based exercise program	12 weeks	Colorectal		Usual activities	71
Knols et al. (2011) [52]	Switzerland	Supervised physical exercise program, with both endurance and resistive strength exercise	12 weeks	Hematopoietic stem-cell transplantation recipients (64.2% lymphoma, 35.9% leukaemia)		Usual care	131
Koevoets et al. (2022) [38] + Witlox et al. (2019) [83]	Netherlands	Aerobic and strength exercise intervention	6 months	Breast (chemotherapy-exposed)	≤ 150 min of moderate-to-vigorous physical activity per week	Usual activities	181
Mardani et al. (2021) [53]	Iran	Exercise program with aerobic, resistant, flexible and pelvic floor muscle exercises	12 weeks	Prostate		Usual care and usual activities	80
Pinto, Frierson, Rabin, Trunzo, & Marcus (2005) [34]	USA	Home-based physical activity intervention, physical activity counselling delivered via telephone + exercise sheets	12 weeks	Breast	Participants should be sedentary	Contact control, usual activities + information but not on PA	86
Pinto, Papandonatos, Goldstein, Marcus, & Farrell (2013) [51]	USA	Home-based telephone counselling physical activity intervention	3 months	Colorectal	Participants should be sedentary	Contact control + information but not on PA	46
Prinsen et al. (2013) [45] + Gielissen, Verhagen, Witjes & Bleijenberg [84]	Netherlands	CBT addressing physical activity	6 months	Any type *(36.5% breast, 23.5% head and neck cancer)*	Participants should be fatigued (severely)	Wait-list	37
Repka & Hayward (2018) [47]	USA	Exercise intervention aerobic + resistance, balance, and flexibility	10 weeks	Any type (radiation or chemotherapy exposed) *(45.6% breast)*	Participants should be sedentary	(1) Usual care + information (2) Non-cancer	22
Rogers et al. (2017) [36] + Rogers et al. (2012) [85]	USA	Physical activity behaviour change intervention	3 months	Breast	Participants should not be regularly active	Usual care + information on PA recommendations	222
Saarto et al. (2012) [30]	Finland	Exercise Program, both supervised and home training	12 months	Breast		Usual activities	573
Short, James, Girgis, D’Souza, & Plotnikoff (2015) [32] *+* Short, James, Girgis, Mcelduff & Plotnikoff 2012) [86] *+* Vallance, Courneya, Taylor, Plotnikoff, & MacKey (2008) [87]	Australia	Tailored-print and targeted-print materials for promoting physical activity, three-arm behaviour change	3 months	Breast	Physical activity readiness (could be active)	Standard recommendation	330
Thorsen et al. (2005) [43]	Norway	Supervised, home-based, training program on physical activity	14 weeks	Breast *(38.0%)*, gynaecologic *(21.5%)*, testicular *(18.0%)* Lymphoma *(22.5%)*		Usual activities	158
Vallance et al. (2020) [40] + Lynch et al. (2018) [88]	Australia	Intervention with wearable technology and behavioural change approaches to increase physical activity and reduce sedentary behaviour	12 weeks	Breast (post menopausal)	Inactive: < 75 min per week of MVPA, and more than seven hours of sedentary behaviour per day	Wait-list control	83
Willems et al. (2017) [44] *+* Willems et al. (2015) [89]	Netherlands	Web-based computer tailored intervention on providing psychological and lifestyle support (mental health, diet, physical activity, fatigue, and other symptoms)	6 months	Any type *(71.2% breast)*		Usual care, wait-list	518
Yun et al. (2020) [48]	South Korea	Health coaching and a web-based program on physical activity, weight, and distress management (three arms)	12 months	Breast *(35.5%)*, Lung *(26.2%)*, Colorectal *(21.6%)* and stomach *(16.8%)*	Participants should not be regularly active	Usual care + health education booklet on PA, diet, and distress management.	394

*For ‘any type’ the percentages of the main one or two cancer types are specified and for multiple types all percentages are presented

^†^Studies had the same baseline characteristics and were consequently analysed as one study [37, 78]

### Characteristics of included studies

The 29 RCTs had a duration that ranged from 4 to 52 weeks (Table 1). The sample size ranged from 22 to 573 participants. Fourteen studies included breast cancer survivors [27–40], eight studies included mixed type of cancer survivors all with the highest percentage of breast cancer survivors [41–48], three studies included colon or colorectal cancer survivors [49–51], one study included people who completed hematopoietic stem-cell transplantation [52], one study included prostate cancer survivors [53], one study included testicular cancer survivors [54], and one study included esophageal cancer survivors [55]. Most (23/29) RCTs were physical activity interventions [28–36, 38–40, 42, 43, 45–47, 49–54] and the other six [27, 37, 41, 44, 48, 55] addressed both physical activity and diet.

Eight studies were classified as high quality [27, 30, 33, 39, 42, 46, 49, 53], eleven studies as acceptable [27, 29, 31, 35–38, 40, 44, 50, 55] and ten studies as low-quality studies [32, 34, 41, 43, 45, 47, 48, 51, 52, 54] (Table 2 overall score and Supplementary material Tables S2a + S2b detailed score). The main reason for a lower quality was the occurrence of differences in characteristics at baseline that could possibly affect fatigue (Table S2b).

**Table 2:** Effects of included trials on fatigue, fatigue type of outcome, questionnaire used for assessing fatigue, and quality of the studies

First author (year)	Outcome type	Questionnaire*	Sig. Effect on fatigue^†^	Quality of study^‡^
Galiano-Castillo (2016) [29]	Not specified/other	R-PFS	Yes	Acceptable
Pinto (2005) [34]	Not specified/other	POMS, Linear analog scale for fatigue	Yes	Low
Adams (2018) [54]	Not specified/other	FACIT-Fatigue Scale	Yes	Low
Koevoets (2022) [38]	Not specified/other	MFI	Yes	Acceptable
Vallance (2020) [40]	Not specified/other	FACIT-Fatigue Scale	Yes	Acceptable
Bantum (2014) [41]	Primary	BFI	No	Low
Cantarero-Villanueva (2012) [27]	Primary	POMS	Yes	High
Cantarero-Villanueva (2013) [28]	Primary	PFS, POMS	Yes	Acceptable
Fillion (2008) [31]	Primary	MFI	No	Acceptable
Kampshoff (2015) [42]	Primary	MFI	Yes	High
Prinsen (2013) [45]	Primary	CIS-Fatigue	Yes	Low
Repka (2018) [47]	Primary	PFI	No	Low
Saarto (2012) [30]	Primary	FACIT-Fatigue Scale	No	High
Willems (2017) [44]	Primary	CIS	Yes	Acceptable
Chang (2020) [55]	Primary (part of QoL)	EORTC-QLQ-C30 fatigue subscale	No	Acceptable
Mardani (2021) [53]	Primary (part of QoL)	EORTC-QLQ-C30 fatigue subscale	Yes	High
Ghavami (2017) [37,78]^§^	Primary, primary (part of QoL)	CFS + EORTC-QLQ-C30 fatigue subscale	Yes	Acceptable
Bennett (2007) [46]	Secondary	Schwartz Cancer Fatigue Scale	No	High
Hartman (2019) [35]	Secondary	PROMIS	No	Acceptable
Kim (2019) [49]	Secondary	FACIT-Fatigue Scale	No	High
Knols (2011) [52]	Secondary	FACT-An Subscale (= FACIT-Fatigue Scale)	No	Low
Pinto (2013) [51]	Secondary	FACIT-Fatigue Scale	No	Low
Short (2015) [32]	Secondary	FACIT-Fatigue Scale	No	Low
Yun (2020) [48]	Secondary	BFI	No	Low
Brown (2018) [50]	Secondary	FSI	Yes	Acceptable
Rogers (2017) [36]	Secondary	FSI	Yes	Acceptable
Thorsen (2005) [43]	Secondary	EORTC-QLQ-C30 fatigue subscale	Yes	Low
Hagstrom (2016) [33]	Secondary	FACIT-Fatigue Scale	Yes	High
Holtdirk (2021) [39]	Secondary	BFI	Yes	High

* FACIT-Fatigue Scale, Functional Assessment of Chronic Illness Therapy – Fatigue Scale; BFI, Brief Fatigue Inventory; (R)-PFS, (Revised) Piper Fatigue Scale; POMS, Profile of Mood States; FSI, Fatigue Symptom Inventory; PROMIS, Patient Reported Outcome Measurement Information System; EORTC-QLQ-C30, European Organisation for Research and Treatment of Cancer Quality-of-life Questionnaire Core 30; MFI, Multidimensional Fatigue Inventory; CFS, Chronic Fatigue Syndrome; FACT-An, Functional Assessment of Cancer Therapy – Anemia; CIS, Checklist Individual Strength -Fatigue

^†^Studies were labelled ‘effective’ when the decrease in fatigue from baseline to post-intervention was statistically significantly (P < 0.05) larger in the intervention group than in the control group

^‡^ Based on the Scottish Intercollegiate Guidelines Network (SIGN) checklist for Randomised controlled trials (20)

^§^ Studies had the same baseline characteristics and were consequently analysed as one study [37, 78]

### Effectiveness of interventions

In total, 17 out of 29 studies were effective in reducing fatigue (Table 2 + 3). Thirteen of the 19 studies classified as high and acceptable quality were effective in reducing fatigue. Twelve out of 29 RCTs had fatigue as primary outcome, twelve studies as secondary outcome, five as ‘other outcome’ (Table 2). In studies with fatigue as primary outcome, seven out of 12 studies effectively reduced fatigue (statistically significant differences between groups). In studies with fatigue as secondary outcome, five out of 12 studies showed an effect on fatigue. The five studies with fatigue as ‘other outcome/not specified outcome’ all showed an effect on fatigue.

### Behaviour change techniques

When considering all studies (n = 29) we observed that 40 out of 93 BCTs were used. When considering only the studies of high and acceptable quality (n = 19), 36 out of 93 BCTs were used (Table 3). The most used BCTs did not differ when examining all studies together and examining only the studies classified as high and acceptable quality. These were Goal setting (behaviour) (n = 25 out of 29 studies in total, n = 16 out of 19 studies of high or acceptable quality), Instruction on how to perform the behaviour (n = 26/29, n = 17/19), Demonstration of the behaviour (n = 21/29, n = 16/19), Behavioural practice/rehearsal (n = 25/29, n = 17/19) and Credible Source (n = 22/29, n = 15/19). Other BCTs in more than half of all studies, but also in studies of high and acceptable quality, include Feedback on behaviour, Self-monitoring of behaviour, Social support (unspecified) and Graded tasks.

**Table 3:**
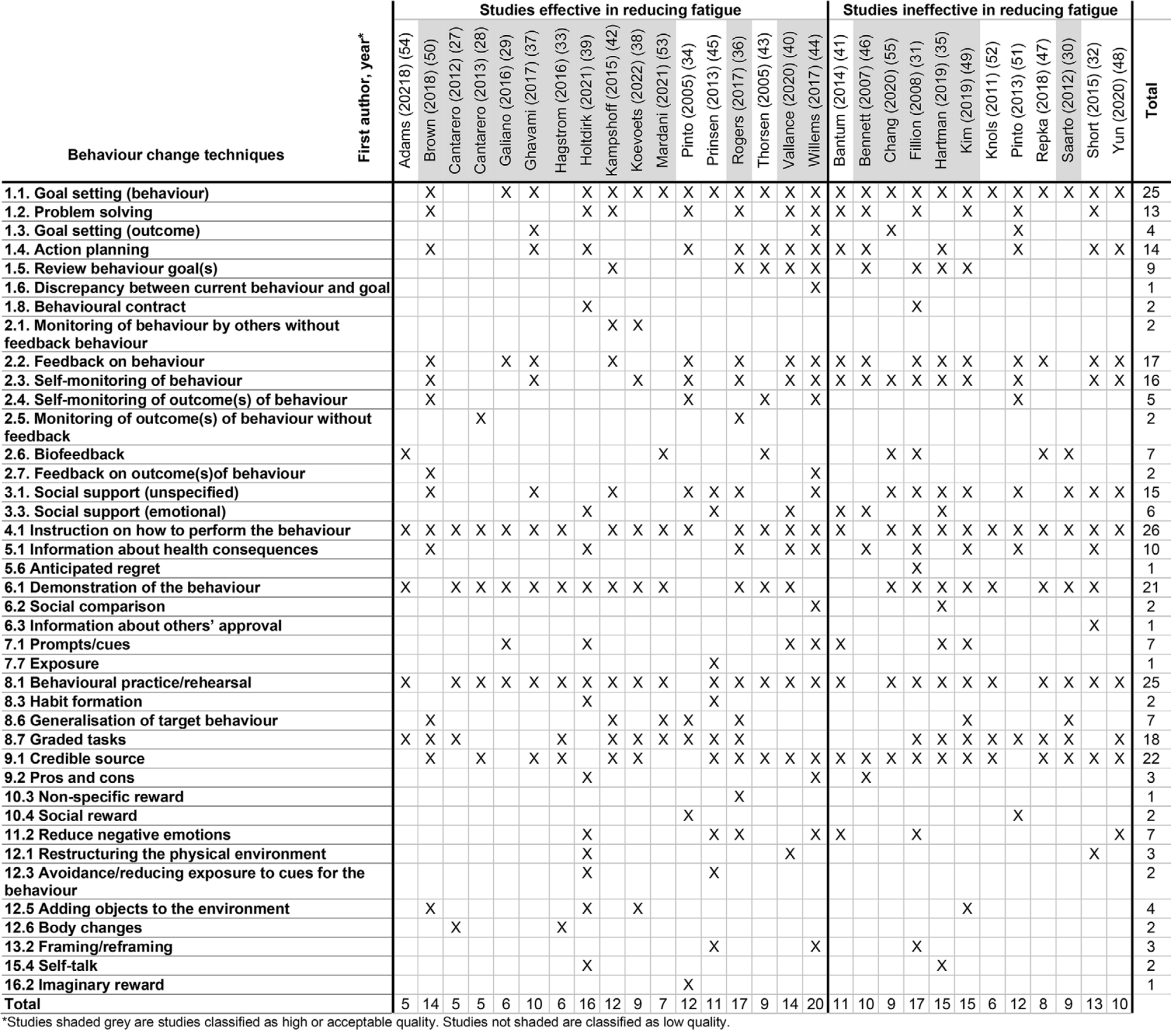
Coded behaviour change techniques in included studies using the BCT Taxonomy [11]

#### Behaviour change techniques in effective vs. ineffective studies

BCTs used in at least 25% of all studies effective in reducing fatigue were Goal setting (behaviour), Problem solving, Action planning, Review behaviour goal(s), Feedback on behaviour, Self-monitoring of behaviour, Social support (unspecified), Instruction on how to perform the behaviour, Information about health consequences, Demonstration of the behaviour, Behaviour practice/rehearsal, Generalisation of the target behaviour, Graded tasks and Credible source (Table 4). These were all also used in at least 25% of studies that were ineffective in changing fatigue, with the notable exception of Generalisation of the target behaviour which was only applied in more than 25% of effective studies.

**Table 4:** Frequency of behaviour change techniques in all trials and in trials of high and acceptable quality, effective versus ineffective in reducing cancer-related fatigue

	All studies (N = 29)				Studies of high and acceptable quality (N = 19)			
Behaviour change techniques*	**Effective studies (n = 17)**		**Ineffective studies (n = 12)**		**Effective studies (n = 13)**		**Ineffective studies (N = 6)**	
	**%**	**N**	**%**	**N**	**%**	**N**	**%**	**N**
1.1. Goal setting (behaviour)	*76.5*	*13*	*100.0*	*12*	*76.9*	*10*	*100.0*	*6*
1.2. Problem solving	*41.2*	*7*	*50.0*	*6*	*46.2*	*6*	*50.0*	*3*
1.3. Goal setting (outcome)	11.8	2	16.7	2	15.4	2	16.7	1
1.4. Action planning	*47.1*	*8*	*50.0*	*6*	*46.2*	*6*	*33.3*	*2*
1.5. Review behaviour goal(s)	*29.4*	*5*	*33.3*	*4*	*30.8*	*4*	*66.7*	*4*
1.6. Discrepancy between current behaviour and goal	5.9	1	0.0	0	7.7	1	0.0	0
1.8. Behavioural contract	5.9	1	8.3	1	7.7	1	16.7	1
2.1. Monitoring of behaviour by others without feedback behaviour	11.8	2	0.0	0	15.4	2	0.0	0
2.2. Feedback on behaviour	*47.1*	*8*	*75.0*	*9*	*53.8*	*7*	*66.7*	*4*
2.3. Self-monitoring of behaviour	*41.2*	*7*	*75.0*	*9*	*46.2*	*6*	*83.3*	*5*
2.4. Self-monitoring of outcome(s) of behaviour	23.5	4	8.3	1	15.4	2	0.0	0
2.5. Monitoring of outcome(s) of behaviour without feedback	11.8	2	0.0	0	15.4	2	0.0	0
2.6. Biofeedback	17.6	3	*33.3*	*4*	7.7	1	*50.0*	*3*
2.7. Feedback on outcome(s)of behaviour	11.8	2	0.0	0	15.4	2	0.0	0
3.1. Social support (unspecified)	*41.2*	*7*	*66.7*	*8*	*38.5*	*5*	*83.3*	*5*
3.3. Social support (emotional)	17.6	3	*25.0*	*3*	15.4	2	*33.3*	*2*
4.1 Instruction on how to perform the behaviour	*88.2*	*15*	*91.7*	*11*	*92.3*	*12*	*83.3*	*5*
5.1 Information about health consequences	*29.4*	*5*	*41.7*	*5*	*38.5*	*5*	*50.0*	*3*
5.6 Anticipated regret	0.0	0	8.3	1	0.0	0	16.7	1
6.1 Demonstration of the behaviour	*76.5*	*13*	*66.7*	*8*	*84.6*	*11*	*83.3*	*5*
6.2 Social comparison	5.9	1	8.3	1	7.7	1	16.7	1
6.3 Information about others’ approval	0.0	0	8.3	1	0.0	0	0.0	0
7.1 Prompts/cues	23.5	4	*25.0*	*3*	*30.8*	*4*	*33.3*	*2*
7.7 Exposure	5.9	1	0	0	0	0	0.0	0
8.1 Behavioural practice/rehearsal	*88.2*	*15*	*83.3*	*10*	*92.3*	*12*	*83.3*	*5*
8.3 Habit formation	11.8	2	0.0	0	7.7	1	0.0	0
8.6 Generalisation of target behaviour	**29.4**	**5**	16.7	2	*30.8*	*4*	*33.3*	*2*
8.7 Graded tasks	*58.8*	*10*	*66.7*	*8*	*53.8*	*7*	*66.7*	*4*
9.1 Credible source	*64.7*	*11*	*91.7*	*11*	*69.2*	*9*	*100.0*	*6*
9.2 Pros and cons	11.8	2	8.3	1	15.4	2	16.7	1
10.3 Non-specific reward	5.9	1	0.0	0	7.7	1	0.0	0
10.4 Social reward	5.9	1	8.3	1	0.0	0	0.0	0
11.2 Reduce negative emotions	23.5	4	*25.0*	*3*	23.1	3	16.7	1
12.1 Restructuring the physical environment	11.8	2	8.3	1	15.4	2	0.0	0
12.3 Avoidance/reducing exposure to cues for the behaviour	11.8	2	0.0	0	7.7	1	0.0	0
12.5 Adding objects to the environment	17.6	3	8.3	1	23.1	3	16.7	1
12.6 Body changes	11.8	2	0.0	0	15.4	2	0.0	0
13.2 Framing/reframing	11.8	2	8.3	1	7.7	1	16.7	1
15.4 Self-talk	5.9	1	8.3	1	7.7	1	16.7	1
16.2 Imaginary reward	5.9	1	0.0	0	0.0	0	0.0	0

* In italic behaviour change techniques that are used in ≥ 25% of interventions. In bold behaviour change technique indicated as a promising technique for reducing cancer-related fatigue, which is a technique used in ≥ 25% of effective interventions, and in < 25% of ineffective studies

When only considering the studies of high and acceptable quality, BCTs that were used in at least 25% of studies effective in reducing fatigue were similar to the ones used in all studies with the addition of Prompts/cues (Table 4). All these techniques were also used in more than 25% of studies that were not effective in changing fatigue.

## Discussion

The main goal of this review was to identify BCTs in interventions that focus specifically on reducing fatigue in cancer survivors who finished treatment, as to the best of our knowledge, this had not been reported on before. We identified 29 RCTs that reported on the effect of lifestyle interventions on fatigue among cancer survivors. Seventeen out of these 29 studies were effective in reducing fatigue and 13 of the 19 studies classified as high and acceptable quality were effective in reducing fatigue. The top five of most applied BCTs were: Goal setting (behaviour), Instruction on how to perform the behaviour, Demonstration of the behaviour, Behavioural practice/rehearsal, and Credible Source. The BCT Generalisation of the target behaviour was identified as ‘promising BCT.’

### Effectiveness of interventions

Seventeen out of 29 RCTs were effective in reducing fatigue, which raises the question why the other studies failed to substantially reduce fatigue. We speculate that five factors may be important to consider.

One, slightly more of the effective studies were classified as having a high or acceptable quality compared with studies that were ineffective in changing fatigue. This may indicate that a RCT that is of robust good quality may be more likely to effectively change fatigue. However, there are important limitations to using tools to evaluate the quality of RCTs that test lifestyle interventions, which we will discuss later.

Two, in our review, studies that had fatigue as primary outcome where more often effective in reducing fatigue than studies with fatigue as secondary outcome. Studies with fatigue as secondary outcome may not have been sufficiently powered to detect a change in fatigue, while the intervention methodology of such trials, including BCTs, is probably based on the primary outcome. Notably, five studies with fatigue as ‘other outcome/not specified outcome’ were all also effective. It is important to specify the outcome type in advance to avoid the inflated risk of false positive findings [56]. As also acknowledged by others, outcomes should be termed exploratory outcomes when they are not a priori reported as primary and secondary outcomes, and the hypothesis generating nature of these should be reported when publishing the results [56].

Three, only three studies included exclusively participants suffering from fatigue [28, 45, 46]. Initially, we aimed to make a comparison of studies with and studies without an inclusion criterion for experiencing a certain level of fatigue to assess whether they differed in effectiveness and the use of BCTs. Due to the limited number of studies that included participants with a certain level of fatigue, this comparison was not possible. If participants did not or hardly experienced fatigue, this limits the possibility to detect an effect on fatigue (i.e., ceiling effect).

Four, it could be that the specific intervention simply did not have an impact on fatigue. And five, it might be that the health behaviours did not sufficiently change to establish a change in fatigue. We attempted to evaluate whether health behaviours were changed by examining between group changes in physical activity and/or dietary intake. However, almost half of the studies did not report estimates on the extend of health behaviour change. Some studies reported adherence rates for attending exercise classes of participants in the intervention group only, or reported outcomes related to physical activity or diet (e.g., VO_2_max, blood markers, physical fitness, or strength). However, due to heterogeneity in the assessment of behaviour change and the lack of reporting of behaviour change effectiveness in other studies, it was not possible to draw conclusions on actual health behaviour change and its effect on the effectiveness of changing fatigue.

### Behaviour change techniques

The BCT ‘Generalisation of the target behaviour’ was used more often in effective studies compared to ineffective studies but was not in our top 10 of most used BCTs, suggesting that interventions could benefit from the application of The BCT ‘Generalisation of the target behaviour’ and increase the chance to effectively reduce fatigue. Compared to other BCTs, little research has been conducted on the link of the BCT ‘Generalisation of the target behaviour’ with specific determinants [57]. Only an inconclusive link was found with improving skills [57]. However, when looking at the studies in our review, we could argue that this BCT may indicate the importance of incorporating change in behaviour in the own environment, and not solely in for example, exercise classes. Many studies focussed on short-term behaviour change by offering exercise classes, but less attention was given to translating/incorporating this behaviour to/in daily life. This was also reflected in the top five of BCTs that were applied as these are mostly techniques typically used in exercise classes. They are often coded together and are often applied by default in exercise classes, that is, receiving instructions and demonstrations from a credible source, such as a physical therapist, who bases instructions on a particular exercise goal toward which the intervention is directed.

However, for new behaviours to be maintained over time, they need to become the dominant response across contexts [58]. Learning theory suggests that long-term maintenance of changed behaviour may be promoted by among others situating the new learning in the most relevant contexts and varying the contexts in which the new learning takes place [59]. Maintenance of behaviour also asks for creating routine and the formation of habitual behaviour [58]. Habit theory argues that repeatedly choosing a behaviour in a stable context can lead to the behaviour becoming automatic over time [60–63], for which it also seems important that the behaviour is thus incorporated into daily life to be maintained after an intervention ends. This is underpinned by the fact that following disruptions in behavioural context, new behaviours might emerge initially, but people still fall back to old patterns as soon as things get back to normal [64]. Studies should therefore provide tools on how to generalize behaviour learned in for example exercise classes to behaviour in real life to maintain behaviour change.

Studies that were effective and studies that were ineffective in reducing fatigue often used the same set of techniques. As also acknowledged by others [65, 66], this makes it difficult to identify promising techniques and suggests that there might be other population or context characteristics that influence effectiveness [66]. For example, it may be that the BCTs that were used were not tailored to population-specific behavioural determinants of behaviour. Many studies did not report links of BCTs to the determinants, i.e., the mechanisms of actions. This might indicate that techniques were used at random and/or without notice. Interventions should provide the rationale for choosing to apply certain BCTs and how they match with population-specific determinants of behaviour. The theory and technique tool [57] can be used to link these BCTs to population-specific behavioural determinants. The combination of BCTs might also have an influence on the effectiveness. This could be because the effects of individual BCTs may be small, BCTs do not often occur separate in an intervention and might also interact with each other [26]. The effectiveness can also differ based on how the BCTs are delivered [26].

In addition, to estimate whether behaviour is successfully maintained after the intervention ends, studies should include a follow-up measurement point after the intervention has ended. In the current review, few (n = 9) studies [27–29, 31, 39, 40, 51, 54, 55] examined this long-term behaviour change, and therefore it was not possible to assess long-term behaviour change.

The coding of the BCTs was sometimes challenging because of four reasons. First, studies were sometimes not sufficient in describing the intervention content. The information given was either too broad or too vague to precisely code the used BCT, which results in low replicability of coding of BCTs [67]. The lack of detail in methodological descriptions, limits the chance of identifying the effective ingredients of interventions [68]. Second, we experienced differences between researchers in coding the BCTs, which is a phenomenon that was also reported in a previous comparable review on BCTs [69]. Again, this is caused by the poor descriptions of the intervention content or the interpretation of the BCTs, its explanation and/or its examples. Third, intervention descriptions vary in terminology used to describe BCTs (i.e., same label applied to different BCTs, and different labels applied to the same BCT) [67]. Fourth, in the examined RCTs it was not always clear if, and which protocol belonged to the RCT or on which information the intervention content was based, which could have led to missing out on some BCTs. Thus, it is urgent that interventions are described more clearly to increase replicability. Investigators should describe the content of the intervention in detail in, for example, a protocol paper and the behaviour change technique taxonomy can be used to indicate which BCT was used.

When considering only the studies of high and acceptable quality, no promising BCTs were identified. These findings on the analysis of studies with high and acceptable quality, should be interpreted with clear caution, as there are important limitations to the use of tools like the SIGN tool [20] to evaluate the quality of RCTs that test lifestyle interventions. One limitation is that there is a level of subjectivity in the interpretation of the quality criteria: different assessors may come to a different conclusion regarding the quality of a trial. Despite the guidance that the tools give to standardize the assessment process, other researchers may not agree with the judgements that we made. Second, assessors can only assess what is reported in papers: when something is not reported or not in enough detail, the quality cannot be assessed. Third, the final assessment of the quality may be different for different outcomes as the weight given to a certain criterion might differ per outcome and research question of the systematic review [70]. Tool designers often recommend showing all answers to each criterion to prevent the expression of the quality of the study in one score only [70], but it is inevitable to categorize the studies into high, acceptable, or low quality to be able to take the quality into account in the analyses. We argue that, although the tools can help to better understand quality aspects of a trial, this categorisation of studies is an oversimplification and may not be reproducible. This also reiterates the point that we made that papers should report details of the study with enough detail to allow for a good assessment of study quality.

### Strengths and limitations

The main strength of our review is that, to the best of our knowledge, this is the first review that examined the use of BCTs in interventions that focus specifically on reducing fatigue in survivors who finished treatment. In addition, in examining promising BCTs we described the difference in BCTs used in effective versus ineffective studies in reducing fatigue, whereas some previous reviews did not evaluate effectiveness or make such a comparison to ineffective studies [21, 22, 24, 71–73].

There are some limitations to discuss. This review retrieved mostly physical activity interventions, which may make it more difficult to extrapolate the findings to lifestyle interventions with both a physical activity and dietary component. The effectiveness of the applied BCTs was assessed via an indirect effect on fatigue through lifestyle changes, but as mentioned before, evaluation of actual behaviour change following the intervention was not possible due to lack of reporting of behaviour change and due to heterogeneity in studies that did report behaviour change. Another limitation was the coding of studies as either effective or ineffective, thereby omitting the effect sizes. We attempted to examine effect sizes rather than p-values, but this was not possible due to heterogeneity across studies in questionnaires used to assess fatigue and in how studies were statistically analysed, as other reviews also pointed out [71, 72]. As the focus of this review was less on actual effectiveness of the intervention, but more on the description of BCTs used in effective versus ineffective studies, we do deem our approach to assess effectiveness of the interventions a plausible method for this cause [21, 22, 24]. Finally, as mentioned before, there is no gold standard yet for determining the effectiveness of BCTs. However, we compared BCTs in the effective studies with BCTs in the ineffective studies, which increases the chance of identifying actual effective BCTs.

### Implications and recommendations for future research

There is a need for studies that screen their participants on level of fatigue and studies that focus more on dietary behaviours to evaluate promising BCTs for reducing fatigue through dietary behaviour change. The insights from this systematic review might help future RCTs to design an effective program for reducing fatigue in cancer survivors, for example, by not only focussing on short-term behaviours learned in for example exercise classes but by also generalising the learned behaviours to the own environment to facilitate long-term behaviour change. This should then be measured by including follow-up time points after the intervention ends. A stepwise approach, such as the Intervention Mapping approach, can be followed for shaping an evidence-based behavioural intervention that is rooted in theory [74]. In addition, future studies should report on whether the behaviour is actually changed. Mediation analyses in RCTs can provide valuable insights in BCTs effective in reducing fatigue through health behaviour change [75].

## Conclusion

Lifestyle interventions that target fatigue in cancer survivors, can apply the BCT ‘Generalisation of the target behaviour’ to incorporate lifestyle behaviours in daily life and increase the chance to effectively reduce fatigue. There is a need for studies that test the effect of dietary interventions on fatigue and a need for studies that screen their participants on level of fatigue. Lifestyle interventions should describe interventions in detail and report on actual behaviour change following the intervention. Long-term behaviour change should be examined by including follow-up timepoints after the interventions ends.

### Electronic supplementary material

Below is the link to the electronic supplementary material.

Supplementary Material 1: Search queries and quality assessment of included studies.

## Data Availability

The datasets during and/or analysed during the current study are available from the corresponding author on reasonable request.

## Abbreviations

BCTs: Behaviour Change Techniques
HRQoL: Health-Related Quality of Life
PICO: Participants, Intervention, Comparison and Outcome
PRISMA: Preferred Reporting Items for Systematic Review and Meta-analysis
RCTs: Randomized Controlled Trials
