# Supplementary material for: Behaviour change techniques used in lifestyle interventions that aim to reduce cancer-related fatigue in cancer survivors: a systematic review
Source: Int J Behav Nutr Phys Act. 2023 Oct 13;20:126. doi: 10.1186/s12966-023-01524-z (PMC10576285; doi:10.1186/s12966-023-01524-z)
Supplement: Supplementary file 1 — Supplementary Material 1: Search queries and quality assessment of included studies. [file 12966_2023_1524_MOESM1_ESM.docx]

*Table S1. Search queries based on the Participants, Intervention, Comparison and Outcome (PICO) framework* (1)*.*

| PubMed | |
| --- | --- |
| 1. Participants | cancer survivors OR recovered cancer patients OR cancer survivorship |
| 2. Intervention | behaviour OR behaviour change OR behaviour change techniques OR behaviour change theory OR behaviour change methods OR behaviour determinants OR behaviour analysis OR psychology intervention OR psychology therapy OR psychological OR psychosocial therapy OR psychosocial intervention |
|  | "randomized controlled trial" OR RCT OR intervention |
|  | lifestyle OR exercise OR physical activity OR sports OR nutrition OR diet* OR "food pattern" OR nutrition science OR nutrition status OR consumption OR eating OR healthy eating OR dietary habits OR "dietary intake" |
| 3. Comparison | *Not used in search term.* |
| 4. Outcome | "cancer-related fatigue" OR "cancer related fatigue" OR fatigue OR CRF OR asthenia OR exhaustion OR tired* OR "loss of energy" |
| Total queries | 1+2+4 (searched in full texts) |
| Scopus | |
| 1. Participants | cancer survivors OR recovered cancer patients OR cancer survivorship |
| 2. Intervention | "behavior*" OR "behavior* change" OR "behavior* change techniques" OR "behavior* change theory" OR "behavior* change methods" OR "behavior* determinants" OR "behavior* analysis" OR "psycholog* intervention" OR "psycholog* therapy" OR "psycholog*" OR psychosocial AND therapy OR psychosocial AND intervention |
|  | "randomized controlled trial" OR rct OR intervention |
|  | lifestyle OR exercise OR physical AND activity OR sports OR nutrition OR "diet*" OR "food pattern" OR "nutrition* science" OR "nutrition* status" OR consumption OR "eat*" OR healthy AND eating OR dietary AND habits OR "dietary intake" |
| 3. Comparison | *Not used in search term.* |
| 4. Outcome | "cancer-related fatigue" OR "cancer related fatigue" OR fatigue OR crf OR asthenia OR exhaustion OR "tired*" OR "loss of energy" |
| PsycINFO | |
| 1. Participants | cancer survivors OR recovered cancer patients OR cancer survivorship |
| 2. Intervention | behavior* OR behaviour* OR behavior* change OR behaviour* change OR behavior* change technique OR behaviour* change technique OR behavior* change theory OR behaviour* change theory OR behavior* change method OR behaviour* change method OR behavior* determinant OR behaviour* determinant OR behavior* analysis OR behaviour* analysis OR psycholog* intervention OR psycholog* therapy OR psycholog* OR psychosocial therapy OR psychosocial intervention |
|  | "randomized controlled trial*" OR rct OR intervention |
|  | lifestyle OR exercise OR physical activity OR sports OR nutrition OR diet* OR "food pattern*" OR nutrition* science OR nutrition* status OR consumption OR eat* OR healthy eating OR dietary habits OR "dietary intake*" |
| 3. Comparison | *Not used in search term.* |
| 4. Outcome | "cancer-related fatigue" OR "cancer related fatigue" OR fatigue OR crf OR asthenia OR exhaustion OR tired* OR "loss of energy” |
| Cochrane Library | |
| 1. Participants | cancer survivor* OR recovered cancer patient* OR cancer survivorship |
| 2. Intervention | behavior* OR behaviour* OR behavior* change OR behaviour* change OR behavior* change technique* OR behaviour* change technique* OR behavior* change theor* OR behaviour* change theor* OR behavior* change method* OR behaviour* change method* OR behavior* determinant* OR behaviour* determinant* OR behavior* analys* OR behaviour* analys* OR psycholog* intervention** OR psycholog* therap* OR psycholog* OR psychosocial therap* OR psychosocial intervention* |
|  | "randomized controlled trial" OR rct OR intervention* |
|  | lifestyle OR exercise OR physical activity OR sport* OR nutrition OR diet* OR "food pattern*" OR nutrition* science* OR nutrition* status OR consumption OR eat* OR healthy eating OR dietary habit* OR "dietary intake" |
| 3. Comparison | *Not used in search term.* |
| 4. Outcome | "cancer-related fatigue" OR "cancer related fatigue" OR fatigue OR crf OR asthenia OR exhaustion OR tired* OR "loss of energy" |
| Web of Science | |
| 1. Participants | cancer survivor* OR recovered cancer patient* OR cancer survivorship |
| 2. Intervention | (behavior* OR behaviour* OR behavior* change OR behaviour* change OR behavior* change technique* OR behaviour* change technique* OR behavior* change theor* OR behaviour* change theor* OR behavior* change method* OR behaviour* change method* OR behavior* determinant* OR behaviour* determinant* OR behavior* analys* OR behaviour* analys* OR psycholog* intervention** OR psycholog* therap* OR psycholog* OR psychosocial therap* OR psychosocial intervention* |
|  | "randomized controlled trial" OR rct OR intervention* |
|  | lifestyle OR exercise OR physical activity OR sport* OR nutrition OR diet* OR "food pattern*" OR nutrition* science* OR nutrition* status OR consumption OR eat* OR healthy eating OR dietary habit* OR "dietary intake" |
| 3. Comparison | *Not used in search term.* |
| 4. Outcome | "cancer-related fatigue" OR "cancer related fatigue" OR fatigue OR crf OR asthenia OR exhaustion OR tired* OR "loss of energy" |
| All databases: to make total queries | **1+2+4 (searched in full texts)** |

Table S2a. Quality assessment of included studies (n=29) based on the Scottish Intercollegiate Guidelines Network (SIGN) checklist for Randomised controlled trials, start questions and internal validity (2).

| Studies | SQ1* | SQ2 | 1.1 | 1.2 | 1.3 | 1.4 | 1.5 | 1.6 | 1.7 | 1.8 | 1.9 | 1.10 |
| --- | --- | --- | --- | --- | --- | --- | --- | --- | --- | --- | --- | --- |
| Adams (2018) (3) | YES | YES | YES | YES | NO | NO | YES | YES | YES | YES | YES | NA |
| Bantum (2014) (4) | YES | YES | YES | YES | NO | NO | NO | YES | YES | YES | CAN'T SAY | NA |
| Bennett (2007) (5) | YES | YES | YES | YES | YES | NO | YES | YES | YES | CAN'T SAY | YES | NA |
| Brown (2018) (6) | YES | YES | YES | YES | YES | NO | CAN'T SAY | YES | YES | CAN'T SAY | YES | NA |
| Cantarero-Villanueva (2012) (7) | YES | YES | YES | YES | YES | NO | YES | YES | YES | YES | YES | NA |
| Cantarero-Villanueva (2013) (8) | YES | YES | YES | YES | YES | NO | YES | YES | YES | YES | NO | NA |
| Chang (2020 (9) | YES | YES | YES | YES | YES | NO | NO | YES | YES | YES | CAN'T SAY | NA |
| Fillion (2008) (10) | YES | YES | YES | YES | YES | NO | NO | CAN'T SAY | YES | YES | YES | NA |
| Galiano-Castillo (2016) (11) | YES | YES | YES | YES | YES | NO | YES | NO | YES | YES | YES | NA |
| Ghavami (2017) (12) | YES | YES | YES | YES | YES | NO | YES | YES | YES | CAN'T SAY | CAN'T SAY | NA |
| Hagstrom (2016) (13) | YES | YES | YES | YES | YES | NO | YES | YES | YES | YES | YES | NA |
| Hartman (2019) (14) | YES | YES | YES | YES | YES | NO | YES | NO | YES | YES | YES | NA |
| Holtdirk et al. (2021) (15) | YES | YES | YES | YES | YES | NO | CAN'T SAY | YES | YES | YES | YES | NA |
| Kampshoff (2015) (16) | YES | YES | YES | YES | YES | NO | YES | YES | YES | YES | YES | CAN'T SAY |
| Kim (2019) (17) | YES | YES | YES | YES | YES | NO | YES | YES | YES | YES | YES | NA |
| Knols (2011) (18) | YES | YES | YES | CAN'T SAY | YES | NO | YES | YES | YES | YES | YES | CAN'T SAY |
| Koevoets et al. (2022) (19) | YES | YES | YES | YES | YES | NO | NO | YES | YES | YES | YES | CAN'T SAY |
| Mardani et al. (2021) (20) | YES | YES | YES | YES | YES | NO | YES | YES | YES | YES | CAN'T SAY | NA |
| Pinto (2005) (21) | YES | YES | YES | CAN'T SAY | NO | NO | YES | YES | YES | YES | YES | NA |
| Pinto (2013) (22) | YES | YES | YES | CAN'T SAY | NO | NO | YES | YES | YES | YES | YES | NA |
| Prinsen (2013) (23) | YES | YES | YES | YES | YES | NO | NO | YES | YES | NO | NO | NA |
| Repka (2018) (24) | YES | YES | YES | NO | NO | NO | NO | YES | YES | YES | CAN'T SAY | NA |
| Rogers (2017) (25) | YES | YES | YES | YES | YES | NO | YES | NO | YES | YES | YES | CAN'T SAY |
| Saarto (2012) (26) | YES | YES | YES | YES | YES | NO | YES | YES | YES | YES | YES | CAN'T SAY |
| Short (2015) (27) | YES | YES | YES | YES | YES | NO | NO | NO | YES | YES | YES | NA |
| Thorsen (2005) (28) | YES | YES | YES | YES | YES | NO | NO | YES | YES | NO | YES | NA |
| Vallance (2020) (29) | YES | YES | YES | YES | YES | NO | NO | YES | YES | YES | YES | NA |
| Willems (2017) (30) | YES | YES | YES | YES | YES | NO | NO | YES | YES | YES | YES | NA |
| Yun (2020) (31) | YES | YES | YES | YES | YES | NO | YES | NO | YES | NO | YES | NA |

*SQ1: Is the paper a Randomised controlled trial or a controlled clinical trial?

SQ2: Is the paper relevant to key question?

1.1: The study addresses an appropriate and clearly focused question.

1.2: The assignment of subjects to treatment groups is randomised.

1.3: An adequate concealment method is used.

1.4: The design keeps subjects and investigators ‘blind’ about treatment allocation. Due to the nature of the studies, blinding of participants is not possible in lifestyle interventions. Therefore, all studies scored a ‘No’ on blinding, and we did not consider this in the final assessment.

1.5: The treatment and control groups are similar at the start of the trial. A ‘NO’ was scored for the following factors when they were different at baseline between groups as these are expected to have an effect on fatigue: age, relationship status, VO2max, physical activity level, cancer stage, treatment, medication for mental health and fatigue level.

1.6: The only difference between groups is the treatment under investigation.

1.7: All relevant outcomes are measured in a standard, valid and reliable way.

1.8: What percentage of the individuals or clusters recruited into each treatment arm of the study dropped out before the study was completed? Scored ‘YES’ when the drop-out was below <20%, scored ‘NO’ when ≥20% and ‘CAN’T SAY’ when the percentage of drop-out was unclear.

1.9: All the subjects are analysed in the groups to which they were randomly allocated (often referred to as intention to treat analysis).

1.10: Where the study is carried out at more than one site, results are comparable for all sites. NA= not applicable

Table S2b. Quality assessment of included studies (n=29) based on the Scottish Intercollegiate Guidelines Network (SIGN) checklist for Randomised controlled trials, overall assessment of the study (2)

| Studies | 2.1 | 2.2 | 2.3 | 2.4 Notes |
| --- | --- | --- | --- | --- |
| Adams (2018) (3) | - | NA | NA |  |
| Bantum (2014) (4) | - | CAN'T SAY | NA |  |
| Bennett (2007) (5) | ++ | NA | YES |  |
| Brown (2018) (6) | + | NA | NA |  |
| Cantarero-Villanueva (2012) (7) | ++ | YES | NA |  |
| Cantarero-Villanueva (2013) (8) | + | YES | YES |  |
| Chang (2020 (9) | + | YES | NA |  |
| Fillion (2008) (10) | + | YES | NA |  |
| Galiano-Castillo (2016) (11) | + | NA | NA |  |
| Ghavami (2017) (12) | + | CAN'T SAY (not based on fatigue) | NA |  |
| Hagstrom (2016) (13) | ++ | NA | NA |  |
| Hartman (2019) (14) | + | NA | NA |  |
| Holtdirk et al. (2021) (15) | ++ | NA | NA |  |
| Kampshoff (2015) (16) | ++ | YES | NA | Concealment method is mentioned, but not sufficiently described. |
| Kim (2019) (17) | ++ | NA | NA |  |
| Knols (2011) (18) | - | NA | NA | Randomisation method is mentioned, but not specified. |
| Koevoets et al. (2022) (19) | + | NA | NA |  |
| Mardani et al. (2021) (20) | ++ | YES | NA |  |
| Pinto (2005) (21) | - | NA | NA | Randomisation method is mentioned, but not specified. |
| Pinto (2013) (22) | - | NA | NA | Randomisation method is mentioned, but not specified. |
| Prinsen (2013) (23) | - | NO | YES |  |
| Repka (2018) (24) | - | CAN'T SAY | NA | Participants were randomised based on date of initial contact (pseudorandomisation). |
| Rogers (2017) (25) | + | NA | NA |  |
| Saarto (2012) (26) | ++ | YES | NA |  |
| Short (2015) (27) | - | NA | NA |  |
| Thorsen (2005) (28) | - | NA | NA |  |
| Vallance (2020) (29) | + | NA | NA |  |
| Willems (2017) (30) | + | CAN'T SAY (not based on fatigue) | NA |  |
| Yun (2020) (31) | - | NA | NA |  |

*2.1 How well was the study done to minimise bias? Codes: High quality (++), Acceptable quality (+), Low quality (-) and Unacceptable – reject (0). Studies were coded based on the internal validity questions (see table S2a) as follows: High quality when there was only one ‘Can’t say’; Acceptable quality when there was one ‘No’ and one ‘Can’t say’ or there were two ‘Can’t say's’; Low quality when there were two till four ‘No’s’; Unacceptable when studies had more than four ‘No’s’. Studies were immediately scored ‘Low quality’ when a ‘No’ or ‘Can’t say’ was scored on either the randomisation or concealment method. Due to the nature of the studies, blinding of participants is not possible in lifestyle interventions. Therefore, all studies scored a ‘No’ on blinding, and we did not consider this in the final assessment.

2.2: Taking into account clinical considerations, your evaluation of the methodology used, and the statistical power of the study, are you certain that the overall effect is due to the study intervention? We estimated whether the studies had reached their intended sample size. This could only be done for the studies that were powered for fatigue (i.e., had fatigue as primary outcome). Since this could not be done for all studies, we did not take this sub-score into account when evaluating the quality of the studies.

2.3: Are the results of this study directly applicable to the patient group targeted by this guideline? We scored all studies that screened their participants on the level of fatigue with ‘YES’, studies that did not screen their participants on fatigue scored ‘NA’ (not applicable). This was done since the effect of the intervention on fatigue can only be generalized to cancer survivors with fatigue. As only three studies screened their participants on the level of fatigue, we did not take this into account when evaluating the quality of the studies.

2.4: Notes. Summarise the authors’ conclusions. Add any comments on your own assessment of the study, and the extent to which it answers your question and mention any areas of uncertainty raised above.

# References

1. Schardt C, Adams MB, Owens T, Keitz S, Fontelo P. Utilization of the PICO framework to improve searching PubMed for clinical questions. BMC Med Inform Decis Mak. 2007;7(1):1–6.

2. Scottish Intercollegiate Guidelines Network (SIGN). Available from URL: http://www.sign.ac.uk. 2023. Methodology Checklist 2: Randomised Controlled Trials. Edinburgh: SIGN; 2023.

3. Adams SC, Delorey DS, Davenport MH, Fairey AS, North S, Courneya KS. Effects of high-intensity interval training on fatigue and quality of life in testicular cancer survivors. Br J Cancer. 2018 May 1;118(10):1313–21.

4. Bantum EOC, Albright CL, White KK, Berenberg JL, Layi G, Ritter PL, et al. Surviving and thriving with cancer using a web-based health behavior change intervention: Randomized controlled trial. J Med Internet Res. 2014;16(2):1–12.

5. Bennett JA, Lyons KS, Winters-Stone K, Nail LM, Scherer J. Motivational interviewing to increase physical activity in long-term cancer survivors: A randomized controlled trial. Nurs Res. 2007;56(1):18–27.

6. Brown JC, Damjanov N, Courneya KS, Troxel AB, Zemel BS, Rickels MR, et al. A randomized dose-response trial of aerobic exercise and health-related quality of life in colon cancer survivors. Psychooncology. 2018;27(4):1221–8.

7. Cantarero-Villanueva I, Fernández-Lao C, del Moral-Avila R, Fernández-de-Las-Peñas C, Feriche-Fernández-Castanys MB, Arroyo-Morales M. Effectiveness of core stability exercises and recovery myofascial release massage on fatigue in breast cancer survivors: A randomized controlled clinical trial. Evidence-based Complementary and Alternative Medicine. 2012;2012.

8. Cantarero-Villanueva I, Fernández-Lao C, Cuesta-Vargas AI, Del Moral-Avila R, Fernández-De-Las-Peñas C, Arroyo-Morales M. The effectiveness of a deep water aquatic exercise program in cancer-related fatigue in breast cancer survivors: A randomized controlled trial. Arch Phys Med Rehabil. 2013;94(2):221–30.

9. Chang YL, Tsai YF, Hsu CL, Chao YK, Hsu CC, Lin KC. The effectiveness of a nurse-led exercise and health education informatics program on exercise capacity and quality of life among cancer survivors after esophagectomy: A randomized controlled trial. Int J Nurs Stud [Internet]. 2020;101:103418. Available from: https://doi.org/10.1016/j.ijnurstu.2019.103418

10. Fillion L, Gagnon P, Leblond F, Gélinas C, Savard J, Dupuis R, et al. A brief intervention for fatigue management in breast cancer survivors. Cancer Nurs. 2008;31(2):145–59.

11. Galiano-Castillo N, Cantarero-Villanueva I, Fernández-Lao C, Ariza-García A, Díaz-Rodríguez L, Del-Moral-Ávila R, et al. Telehealth system: A randomized controlled trial evaluating the impact of an internet-based exercise intervention on quality of life, pain, muscle strength, and fatigue in breast cancer survivors. Cancer. 2016;122(20):3166–74.

12. Ghavami H, Akyolcu N. The Impact of Lifestyle Interventions in Breast Cancer Women after Completion of Primary Therapy: A Randomized Study. Journal of Breast Health. 2017;13(2):94–9.

13. Hagstrom AD, Marshall PWM, Lonsdale C, Cheema BS, Fiatarone Singh MA, Green S. Resistance training improves fatigue and quality of life in previously sedentary breast cancer survivors: a randomised controlled trial. Eur J Cancer Care (Engl). 2016;25(5):784–94.

14. Hartman SJ, Weiner LS, Nelson SH, Natarajan L, Patterson RE, Palmer BW, et al. Mediators of a physical activity intervention on cognition in breast cancer survivors: Evidence from a randomized controlled trial. JMIR Cancer. 2019;5(2):1–15.

15. Holtdirk F, Mehnert A, Weiss M, Mayer J, Meyer BR, Bröde P, et al. Results of the Optimune trial: A randomized controlled trial evaluating a novel Internet intervention for breast cancer survivors. PLoS One. 2021 May 1;16(5 May).

16. Kampshoff CS, Chinapaw MJM, Brug J, Twisk JWR, Schep G, Nijziel MR, et al. Randomized controlled trial of the effects of high intensity and low-to-moderate intensity exercise on physical fitness and fatigue in cancer survivors: results of the Resistance and Endurance exercise After ChemoTherapy (REACT) study. BMC Med. 2015;13(1):275.

17. Kim JY, Lee MK, Lee DH, Kang DW, Min JH, Lee JW, et al. Effects of a 12-week home-based exercise program on quality of life, psychological health, and the level of physical activity in colorectal cancer survivors: a randomized controlled trial. Supportive Care in Cancer. 2019;27(8):2933–40.

18. Knols RH, De Bruin ED, Uebelhart D, Aufdemkampe G, Schanz U, Stenner-Liewen F, et al. Effects of an outpatient physical exercise program on hematopoietic stem-cell transplantation recipients: A randomized clinical trial. Bone Marrow Transplant. 2011;46(9):1245–55.

19. Koevoets EW, Schagen SB, de Ruiter MB, Geerlings MI, Witlox L, van der Wall E, et al. Effect of physical exercise on cognitive function after chemotherapy in patients with breast cancer: a randomized controlled trial (PAM study). Breast Cancer Research. 2022 Dec 1;24(1).

20. Mardani A, Pedram Razi S, Mazaheri R, Haghani S, Vaismoradi M. Effect of the exercise programme on the quality of life of prostate cancer survivors: A randomized controlled trial. Int J Nurs Pract. 2021 Apr 1;27(2).

21. Pinto BM, Frierson GM, Rabin C, Trunzo JJ, Marcus BH. Home-based physical activity intervention for breast cancer patients. Journal of Clinical Oncology. 2005;23(15):3577–87.

22. Pinto BM, Papandonatos GD, Goldstein MG, Marcus BH, Farrell N. Home-based physical activity intervention for colorectal cancer survivors. Psychooncology. 2013;22(1):54–64.

23. Prinsen H, Bleijenberg G, Heijmen L, Zwarts MJ, Leer JWH, Heerschap A, et al. The role of physical activity and physical fitness in postcancer fatigue: A randomized controlled trial. Supportive Care in Cancer. 2013;21(8):2279–88.

24. Repka CP, Hayward R. Effects of an Exercise Intervention on Cancer-Related Fatigue and Its Relationship to Markers of Oxidative Stress. Integr Cancer Ther. 2018;17(2):503–10.

25. Rogers LQ, Courneya KS, Anton PM, Verhulst S, Vicari SK, Robbs RS, et al. Effects of a multicomponent physical activity behavior change intervention on fatigue, anxiety, and depressive symptomatology in breast cancer survivors: randomized trial. Psychooncology. 2017;26(11):1901–6.

26. Saarto T, Penttinen HM, Sievänen H, Kellokumpu-Lehtinen PL, Hakamies-Blomqvist L, Nikander R, et al. Effectiveness of a 12-month exercise program on physical performance and quality of life of breast cancer survivors. Anticancer Res. 2012;32(9):3875–84.

27. Short CE, James EL, Girgis A, D’Souza MI, Plotnikoff RC. Main outcomes of the Move More for Life Trial: A randomised controlled trial examining the effects of tailored-print and targeted-print materials for promoting physical activity among post-treatment breast cancer survivors. Psychooncology. 2015;24(7):771–8.

28. Thorsen L, Skovlund E, Strømme SB, Hornslien K, Dahl AA, Fosså SD. Effectiveness of physical activity on cardiorespiratory fitness and health-related quality of life in young and middle-aged cancer patients shortly after chemotherapy. Journal of Clinical Oncology. 2005;23(10):2378–88.

29. Vallance JK, Nguyen NH, Moore MM, Reeves MM, Rosenberg DE, Boyle T, et al. Effects of the ACTIVity And TEchnology (ACTIVATE) intervention on health-related quality of life and fatigue outcomes in breast cancer survivors. Psychooncology. 2020 Jan 1;29(1):204–11.

30. Willems RA, Bolman CAW, Mesters I, Kanera IM, Beaulen AAJM, Lechner L. Short-term effectiveness of a web-based tailored intervention for cancer survivors on quality of life, anxiety, depression, and fatigue: randomized controlled trial. Psychooncology. 2017;26(2):222–30.

31. Yun YH, Lim C Il, Lee ES, Kim YT, Shin KH, Kim YW, et al. Efficacy of health coaching and a web-based program on physical activity, weight, and distress management among cancer survivors: A multi-centered randomised controlled trial. Psychooncology. 2020;29(7):1105–14.
